# Ammonium Ion-Pre-Intercalated MnO_2_ on Carbon Cloth for High-Energy Density Asymmetric Supercapacitors

**DOI:** 10.3390/ma17081858

**Published:** 2024-04-17

**Authors:** Chaoyi Zheng, Xiaohong Sun, Xinqi Zhao, Xi Zhang, Jiawei Wang, Zhuang Yuan, Zhiyou Gong

**Affiliations:** Key Laboratory of Advanced Ceramics and Machining Technology of Ministry of Education, School of Materials Science and Engineering, Tianjin University, Tianjin 300072, China; 2021208185@tju.edu.cn (C.Z.); 2021208039@tju.edu.cn (Z.Y.);

**Keywords:** manganese dioxide, ammonium ions, pre-intercalation, carbon cloth, asymmetric supercapacitor, high energy density

## Abstract

With the continuous development of green energy, society is increasingly demanding advanced energy storage devices. Manganese-based asymmetric supercapacitors (ASCs) can deliver high energy density while possessing high power density. However, the structural instability hampers the wider application of manganese dioxide in ASCs. A novel MnO_2_-based electrode material was designed in this study. We synthesized a MnO_2_/carbon cloth electrode, CC@NMO, with NH_4_^+^ ion pre-intercalation through a one-step hydrothermal method. The pre-intercalation of NH_4_^+^ stabilizes the MnO_2_ interlayer structure, expanding the electrode stable working potential window to 0–1.1 V and achieving a remarkable mass specific capacitance of 181.4 F g^−1^. Furthermore, the ASC device fabricated using the CC@NMO electrode and activated carbon electrode exhibits excellent electrochemical properties. The CC@NMO//AC achieves a high energy density of 63.49 Wh kg^−1^ and a power density of 949.8 W kg^−1^. Even after cycling 10,000 times at 10 A g^−1^, the device retains 81.2% of its capacitance. This work sheds new light on manganese dioxide-based asymmetric supercapacitors and represents a significant contribution for future research on them.

## 1. Introduction

Energy storage technology holds a crucial position in the technological transformation of modern society. With the speedy growth of renewable energy and electrification technologies, there is a rising demand for novel devices with high efficiency, quick response times, and reliability [1,2,3]. In this context, supercapacitors (SCs) have garnered widespread attention as highly promising energy storage device. SCs offer a fast charge/discharge capability, extended service life, and great power density [4,5,6]. However, conventional supercapacitors still face limitations in terms of energy density. To address this challenge, the concept of asymmetric supercapacitors (ASCs) has emerged [7,8]. ASCs combine the characteristics of electrochemical and double-layer capacitors, aiming to deliver higher energy density, along with high power density. The two electrodes in ASCs are typically divided into battery-type and capacitor-type electrodes. Enhancing the electrochemical behavior of battery-type electrodes is the primary focus of ASC research due to its significant contribution to the high energy density of ASCs [9,10,11].

Among the substrate materials for ASC electrodes, carbon cloth (CC) is a non-metallic substrate with a large specific surface area and outstanding mechanical properties. In contrast to metallic substrates, CC can be stabilized in acidic and alkaline electrolytes [12]. However, carbon cloth has a series of disadvantages, such as its hydrophobicity and poor adhesion of active substances. Therefore, the CC needs to be pretreated to improve its adhesive performance. Pretreated CC is an ideal substrate material for ASC electrodes and can easily load pseudo-capacitance materials using synthesis methods such as the hydrothermal method [13].

Metal oxides are widely used as ASC electrode materials. Among the various metal oxides, manganese dioxide is an electrode material deserving of attention because of its excellent energy storage performance and low cost. Its high specific surface area and abundant controllable redox reactions have garnered a lot of focus in research on ASC devices [14]. However, MnO_2_ has defects such as its poor electronic conductivity and unstable structure, which hinder its further development. Improving the microstructure has been the focus of MnO_2_-based electrode research. There is a variety crystal structures of MnO_2_, such as α, β, γ, δ, etc. Unlike the chain/tunneling-type structures possessed by α, β, and γ-MnO_2_, the layered structure of δ-MnO_2_ provides more possibilities for improving the electrochemical performance of MnO_2_ [15].

Pre-intercalation technology has received much attention as a strategy that can significantly enhance the electrochemical behavior of materials [16]. By pre-intercalating ions into the layered structure, it is possible to stabilize the crystal structure, optimize its electronic conduction properties, and improve responsiveness to electrons. The pre-intercalation technique that has been reported so far mainly involves Na^+^, K^+^, Cu^2+^, Zn^2+^, Al^3+^, and Sn^4+^ [17,18,19,20,21,22,23,24]. Compared with these ions, NH_4_^+^ has a lower molar mass, which can improve the mass specific capacity of MnO_2_ materials. NH_4_^+^-pre-intercalated vanadium oxides have been used in energy storage applications, and the NH_4_^+^ ions contribute to facilitating the fast kinetics of ions/electrons and enhancing structural stability [25]. However, no studies about ammonium ion-pre-intercalated manganese oxides for ASCs have been reported.

**Figure 1:**
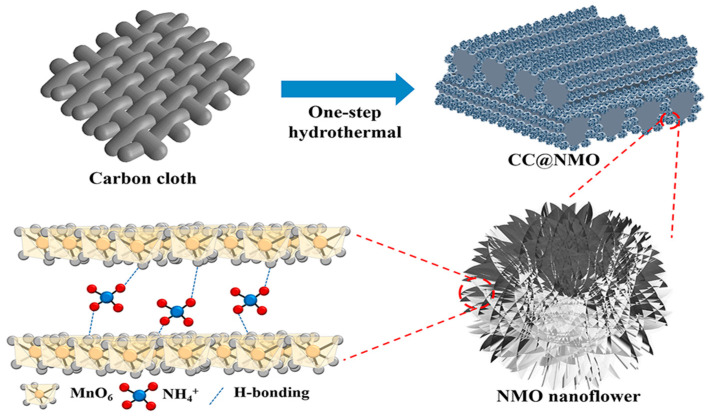
Schematic diagram of the synthesis of CC@NMO.

In this study, we synthesized a manganese dioxide/carbon cloth electrode with ammonium ion pre-intercalation (CC@NMO) using a one-step hydrothermal method, as shown in Figure 1. Via NH_4_^+^ pre-intercalation, the potential window of CC@NMO can be widened from 0–0.8 V to 0–1.1 V against the pure MnO_2_/carbon cloth (CC@MnO_2_) electrode. The CC@NMO electrode was found to have a remarkable capacitance of 181.4 F g^−1^. After being matched with the activated carbon electrode, the CC@NMO electrode was fabricated into a lithium-ion asymmetric supercapacitor (CC@NMO//AC). The energy density of CC@NMO//AC can reach 63.49 Wh kg^−1^, with a power density of 949.8 W kg^−1^. Furthermore, under a high current density of 20 A g^−1^, the energy density can be kept at 31 Wh kg^−1^. After complete cycling for 10,000 cycles at 10 A g^−1^, there is still 81.2% capacitance retention. This work explores the effective improvement of the capacitance properties of MnO_2_ through NH_4_^+^ ion pre-intercalation technology, presenting a new future for high-energy density ASCs.

## 2. Materials and Methods

### 2.1. Pretreatment of CC

The carbon cloth (CC) was cut into 3 cm × 4 cm rectangles and cleaned using deionized water (DIW). The cleaned rectangular CC was ultrasonicated in an acetone solution for 10 min, then ultrasonicated with ethanol for another 10 min; this procedure was repeated three times. Finally, the pretreated CC was put into an oven for drying overnight at 60 °C. Due to the pretreatment and activation of CC, more active sites appeared around the surface of the carbon fibers, enhancing its adhesion to the MnO_2_ [17].

### 2.2. Synthesis of CC@NMO

KMnO_4_ (0.237 g, 1.5 mmol) and CH_3_COONH_4_ (0.038 g, 0.5 mmol) were dissolved in 30 mL DIW and stirred until completely mixed. Then, the pretreated CC was transferred to a 50 mL solution with a Teflon-lined autoclave. The hydrothermal reaction was carried out at 120 °C, lasting for 3 h. After being rinsed with DIW and ethanol, the CC@NMO electrode was cut into 1 cm × 1 cm. The mass loading of the CC@NMO electrode should be 1 mg on a slice of CC with an area of 1 cm^2^.

### 2.3. Fabrication of CC@NMO//AC ASC Device

To fabricate an ASC device, CC@NMO is used as a cathode, with activated carbon (AC) serving as an anode. Depending on charge conservation, there is a need for an exact match between cathode and anode. The mass ratio of both electrodes is estimated using the following:

(1) $$\frac{m_{+}}{m_{-}}=\frac{C_{-}\cdot {∆V}_{-}}{C_{+}\cdot {∆V}_{+}}$$

in which C represents the mass specific capacitance (F g^−1^); ∆V stands for the stably working potential window (V). The mass loading of the AC electrode should be 2 mg, according to Equation (1). After matching the electrodes, an ASC device was fabricated with the two electrodes and 1 M Li_2_SO_4_ solution as the electrolyte.

The AC electrode was prepared as follows: active carbon (AC), Super P, and polyvinylidene fluoride (PVDF) binder in a weight ratio of 7:2:1 were dispersed in N-methylpyrrolidone (NMP) solvent. According to the electrode match, the loading mass of AC should be 2 mg. After stirring, the homogeneous slurry was coated uniformly on a copper foil. After vacuum-drying, it was cut into discs to be used as the electrodes. Then, CR2032-type coin cells were assembled with the two electrodes, glass fiber as a separator, and 1 M Li_2_SO_4_ solution as the electrolyte. After all the processes above, the two-electrode ASC device was finally fabricated.

### 2.4. Characterization

Scanning electron microscopy (SEM) images were obtained using a Zeiss sigma-300 thermal field emission scanning electron (Zeiss, Oberkochen, Germany) microscope to characterize the surface micromorphology of the samples. The existence of elements in the material was characterized using an energy dispersion spectrometer (EDS). To characterize the crystal structures of the samples, their X-ray diffraction (XRD) patterns were obtained using a Bruker D8 Advance (Bruker, Bremen, Germany) using Cu Kα radiation (λ = 0.15406 nm) with 2θ degree ranging from 10° to 80°. Fourier-Transform Infrared Spectroscopy (FTIR, Thermo Fisher, Waltham, MA, USA) was used to reflect the chemical constitution and crystalline structure, with the wavenumber ranging from 4000 to 400 cm^−1^. X-ray photoelectron spectroscopy (XPS, Axis Supra, and Kratos, Manchester, UK) was conducted to further verify the elements’ existence and evaluate their valence states.

### 2.5. Electrochemical Measurements

To evaluate the electric behavior of the CC@NMO, we assembled a three-electrode system for electrochemical testing utilizing a saturated calomel electrode, along with a platinum electrode. Cyclic voltammetry (CV) experiments and galvanostatic charge/discharge (GCD) experiments were carried out by using a CHI660e workstation with 1 M Li_2_SO_4_ as the electrolyte. The capacitance of the CC@NMO electrode can be determined through the GCD curves using the following equation:

(2) $$C=\frac{I\cdot \Delta t}{m\cdot \Delta V}\;(F\cdot g^{-1})$$

in which I denotes the current density (A), ∆t represents the time used for discharging (s), m denotes the material weight, and ∆V stands for the voltage window for the GCD test (V).

To further demonstrate the contribution to the high capacity and energy density of CC@NMO, an ASC device was fabricated using CC@NMO as the cathode and AC as the anode. The CV experiments and GCD experiments were also carried out by using a CHI660e electrochemical workstation. The mass specific capacitance of ASC could be obtained from the GCD curve based on Equation (2). The formulas for calculating the energy density and power density of ASC are as follows:

(3) $$E=\frac{1}{2}\cdot \frac{1}{3.6}C\cdot V^{2}\;(\mathrm{Wh}\cdot {kg}^{-1})$$

(4) $$P=\frac{E}{t}\cdot 3600\;(W\cdot {kg}^{-1})$$

where C denotes the mass specific capacitance of ASC (F g^−1^), V represents the voltage range of ASC (V), and t is the time used for discharging (s) [7].

## 3. Results

**Figure 2:**
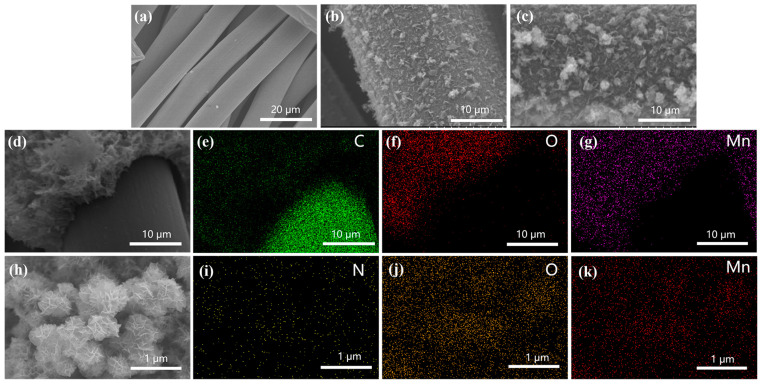
Morphological analyses: (**a**) pretreated CC; (**b**) CC@MnO_2_; (**c**) CC@NMO; (**d**) low magnification of CC@NMO; (**e**–**g**) EDS mapping of C, O, and Mn; (**h**) high magnification of CC@NMO; and (**i**–**k**) EDS mapping of N, O, and Mn.

The SEM images are displayed in Figure 2, revealing the morphology of CC@NMO. From Figure 2a, it can be seen that after ultrasonic treatment in acetone, the surface of the carbon cloth is visible. The wrinkles on the carbon fibers provide active sites for the adhesion of MnO_2_. Figure 2b shows the morphology of CC@MnO_2_, and it can be seen that there are layered MnO_2_ nanosheets on the carbon cloth fibers. Additionally, there are some MnO_2_ nanoflowers, which are assembled from the MnO_2_ nanosheets. The carbon cloth and MnO_4_^−^ ions react as follows: 4MnO_4_^−^ + 3C + 4H^+^ → 4MnO_2_ + 2H_2_O + 3CO_2_↑. This promotes the transition from KMnO_4_ to MnO_2_ [26]. Figure 2c displays the SEM image of CC@NMO. After NH_4_^+^ pre-intercalation, the CC@NMO can retain the morphology without structural collapse. The NMO nanoflowers are clearly recognizable, as are the layered nanosheets. An image of the MnO_2_-covered part and the uncovered part in CC@NMO is displayed in Figure 2d. It is evident that a δ-MnO_2_ layer with a complex laminar structure has been loaded. Figure 2e–g show the results of our EDS elemental mapping analysis of this region, corresponding to the elements C, O, and Mn. It can be inferred that MnO_2_ nanoflowers form a coating structure around the surface of the CC. Figure 2h presents the high magnification of MnO_2_ nanoflowers with a diameter of 500 nm. The nanoflowers are assembled from two-dimensional (2D) ultra-thin nanosheets. This unique structure provides abundant active sites for energy storage, which help the CC@NMO sample to obtain a larger specific capacitance [27]. Through EDS elemental mapping analysis, as shown in Figure 2i–k, the nitrogen element is uniformly distributed around the NMO nanoflowers. TEM and HRTEM images are shown in Figure S1. Figure S1a indicates that the NMO nanoflowers are assembled from layered 2D nanosheets. Moreover, from the HRTEM image (Figure S1b), the distinct lattice fringes of 0.7 nm are assigned to the (001) lattice planes of NMO. All of the above evidence that the NH_4_^+^ ions are successfully intercalated into NMO, with no structure being destroyed.

**Figure 3:**
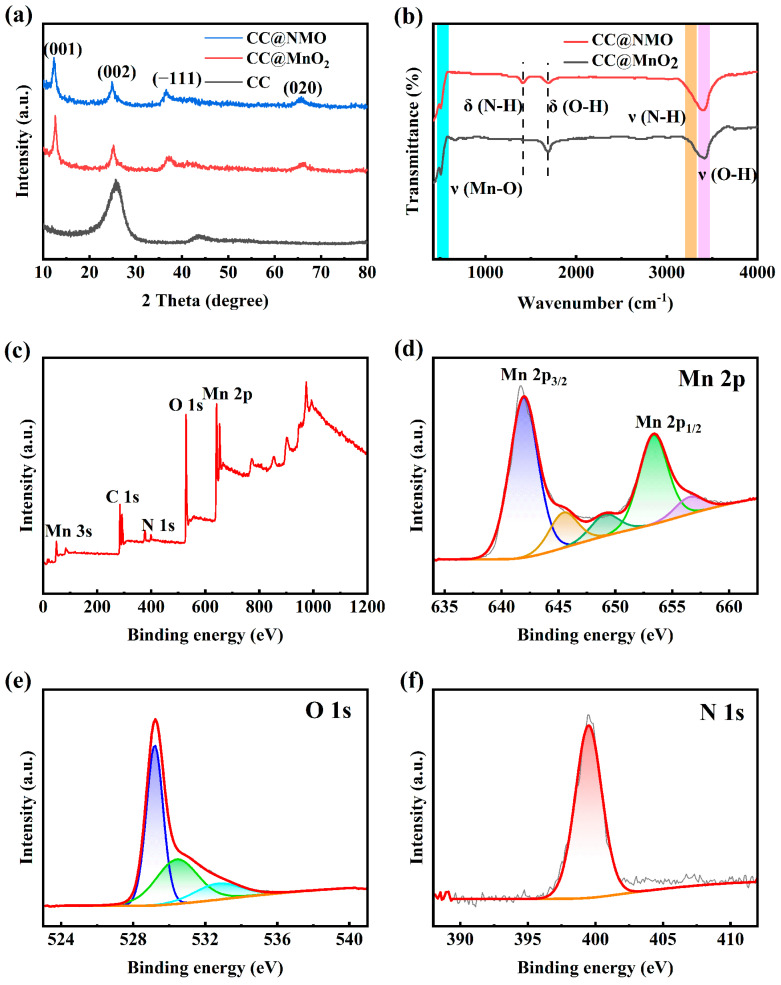
(**a**) XRD patterns of CC@NMO, CC@MnO_2_, and CC; (**b**) FTIR spectra of CC@NMO and CC@MnO_2_; and (**c**) XPS spectra of CC@NMO, (**d**) Mn 2p, (**e**) O 1s, and (**f**) N 1s.

X-ray diffraction (XRD) can characterize the phase concentration and crystal structure. The XRD spectra of CC@MnO_2_, CC@NMO, and CC are shown in Figure 3a. Compared with CC, there are obvious diffraction peaks of CC@MnO_2_ and CC@NMO. The obvious peaks located around 12.5°, 25°, 37.1°, and 65.5° precisely satisfy the peaks of δ-MnO_2_ (JCPDS: 80-1098), which represent (001), (002), (-111), and (020) crystal planes, respectively [27]. The four main characteristic peaks, as well as the absence of other impurity peaks, indicate the successful synthesis of MnO_2_ loaded onto CC. Notably, the (001) peak of the CC@NMO sample has a smaller degree than that of the CC@MnO_2_ sample, as shown in Figure S2. According to Bragg’s Law, the CC@NMO obviously has larger interplanar spacing. This phenomenon is consistent with that of MnO_2_ electrodes pre-intercalated with other ions. The (001) plane of MnO_2_ is our main research subject. Due to the intercalation of NH_4_^+^, the layer spacing of CC@NMO has been expanded. All of these make the CC@NMO capable of providing a larger space for accelerating ion/electron kinetics [28].

Fourier-Transform Infrared Spectroscopy (FTIR) was used to further investigate how NH_4_^+^ intercalates into MnO_2_. Figure 3b displays the FTIR spectra of CC@NMO and CC@MnO_2_. The lattice vibration of Mn-O can be seen between 400 cm^−1^ and 500 cm^−1^, indicating the successful loading of MnO_2_ [29]. The peaks appearing near 1670 cm^−1^ and 3400 cm^−1^ correspond to O-H bending/stretching vibration modes. This phenomenon proves the existence of constitution water molecules between the MnO_2_ layers. Interestingly, it has been proven that the water molecules between the layers can reduce the effect of positive ion electrostatic repulsion, thus promoting the positive ion migration rate [30]. The existence of hydrogen bonding can also increase the charge density of the electrode material [31]. From the spectrum, we can observe that compared with CC@MnO_2_, there is a characteristic peak at 1409 cm^−1^ for CC@NMO, which belongs to the N-H bending mode. The peak around 3200 cm^−1^, corresponding to the hydrogen bonding of N-H, indicates the characteristic N-H···O stretching vibration [32]. All the above demonstrates how NH_4_^+^ ions and water molecules exist in the interlayers of the CC@NMO samples. As interlayer structural columns, NH_4_^+^ ions improve the structural stability of MnO_2_. They can allow the interlayer spacing to expand, accelerating the transport of the ions. Additionally, the water molecules also increase the electrochemical rate of the CC@NMO electrode. Overall, the unique interlayer structure provides strong support for raising the energy density of ASCs [33].

The elemental components and chemistry information of a sample can be provided through X-ray photoelectron spectroscopy (XPS). Figure 3c shows the survey spectrum of CC@NMO, with C, O, Mn, and N elements clearly existing. From the spectrum of Mn2p (Figure 3d), the peaks located at 642 and 653.7 eV can be clearly seen. They are assigned to Mn 2p^3/2^ and Mn 2p^1/2^, respectively [34]. The two peaks are separated by a difference of 11.7 eV, satisfying the characteristic of Mn^4+^ in MnO_2_. Figure 3e is the O 1s spectrum, and the 529.2 eV, 530.4 eV, and 533.0 eV characteristic peaks correspond to Mn-O-Mn, Mn-O-H, and H-O-H bonds, respectively [35,36]. Figure 3f shows the spectra of N 1 s, and the binding energy characteristic peak at 399.6 eV further proves the N element exists. Through all of the above, the existence of NH_4_^+^ ions can be clearly verified, and they are successfully pre-intercalated into the interlayer of MnO_2_ [33].

**Figure 4:**
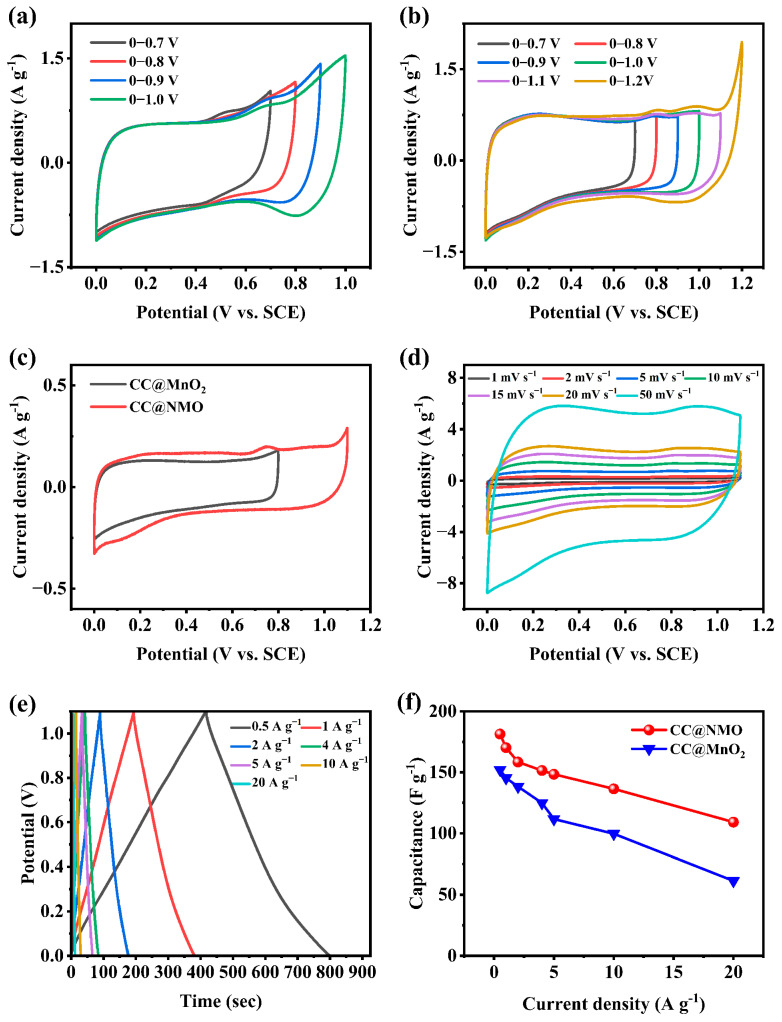
(**a**) CV curves of CC@MnO_2_ at different potential windows at 5 mV s^−1^, (**b**) CV curves of CC@NMO at different potential windows at 5 mV s^−1^, (**c**) comparison of CC@NMO and CC@MnO_2_ at 1 mV s^−1^, (**d**) CV curves of CC@NMO electrode at different scan rates, (**e**) GCD curves of CC@NMO electrode at different current densities, and (**f**) specific capacitance comparison of CC@NMO electrode and CC@MnO_2_ electrode at different current densities.

To test the electrochemical behavior of the CC@NMO, it is necessary to confirm the electrode’s stable working potential window by cyclic voltammetry (CV) testing [37]. Figure 4a depicts the CV curves of CC@MnO_2_ at a range of potential windows under 5 mV s^−1^. The CV shape of CC@MnO_2_ remains approximately rectangular at 0–0.8 V. The peak polarization is more obvious when the potential window exceeds 0.9 V, indicating that the CC@MnO_2_ electrode only works stably in the range of 0–0.8 V [38]. The stable working potential window of the pure MnO_2_ electrode under the neutral aqueous electrolyte is consistent with previous studies [39,40]. In contrast, the CV curves of CC@NMO are at enlarged potential windows at 5 mV s^−1^ (as shown in Figure 4b), which keeps approximately rectangular until it exceeds 1.1 V. After NH_4_^+^ pre-intercalation, the stable working potential window of the CC@NMO electrode was significantly escalated to 0–1.1 V. Interestingly, this phenomenon is consistent with that of other ion-pre-intercalated MnO_2_ electrodes [41]. Due to the structural support provided by NH_4_^+^, the structural stability of NMO has been greatly enhanced [25]. Therefore, the CC@NMO electrode can maintain stability at a larger potential window, which makes it possible to fabricate a higher-energy density ASC.

Figure 4c shows a comparison of the CV behavior of both samples at 1 mV s^−1^. Notably, CC@NMO has redox peaks at 0.15 V and 0.75 V, whereas the CC@MnO_2_ electrode exhibits approximately rectangular peaks. The CC@NMO’s pseudo-capacitance behavior provides a more adequate chemical reaction at the electrode, leading to a larger CV curve area and enhancing the specific capacitance [42]. As depicted in Figure 4d, the shapes of the CV curves are approximately ideal rectangles at different scan rates. Even at a high scan rate, the CC@NMO electrode still maintains a fast ion diffusion rate and exhibits great capacitance performance. The EIS curves of CC@MnO_2_ and CC@NMO in a three-electrode system are shown in Figure S3. For the two electrodes, the EIS curves represent a minor semicircle in the high-frequency section and an almost vertical diagonal line in the low-frequency section. The equivalent series resistance value of 2.54 Ω of the CC@NMO electrode is much lower than CC@MnO_2_ (3.51 Ω). Additionally, the slope of the CC@NMO electrode in the low-frequency section is steeper than that of the CC@MnO_2_ electrode, which indicates that the electrolyte has a better diffusion behavior on the CC@NMO electrode. The EIS curves reveal that CC@NMO has faster charge transfer and ion diffusion because of NH_4_^+^ pre-intercalation.

The GCD curves of the CC@NMO electrode (shown in Figure 4e) reflect the capacity performance. The charging and discharging time can be as long as 390 s at 1 A g^−1^, which indicates that CC@NMO has excellent capacitive behavior. Moreover, the triangle-like GCD curves can maintain their shape with the gradual increase in current density. Meanwhile, the stable triangular GCD curves mean the electrode has excellent charge/discharge stability and good reversibility [28,43]. The GCD curves of CC@MnO_2_ in three-electrode systems are shown in Figure S4. The mass specific capacitance can be calculated by Equation (2). As displayed in Figure 4f, at 1 A g^−1^, the capacitance of CC@NMO is as high as 170.17 F g^−1^. The capacitance of the CC@NMO electrode can still remain at 109.09 F g^−1^ at 20 A g^−1^. At a current density of 0.5 A g^−1^, the capacitance of CC@MnO_2_ is 126.7 F g^−1^, which is much lower than the capacitance of CC@NMO (181.4 F g^−1^). The cycling test of CC@NMO under a current density of 5 A g^−1^ in a three-electrode system is shown in Figure S5. For the first four cycles, the capacitance of CC@NMO is 147 F g^−1^. After 500 cycles, the capacitance of CC@NMO can remain 145.65 F g^−1^. The CC@NMO shows excellent cycle stability, with a very low capacitance loss. Obviously, the CC@NMO electrode has extraordinary capacitance performance and charging/discharging stability. In general, the CC@NMO electrode is an excellent choice for asymmetric supercapacitors.

**Figure 5:**
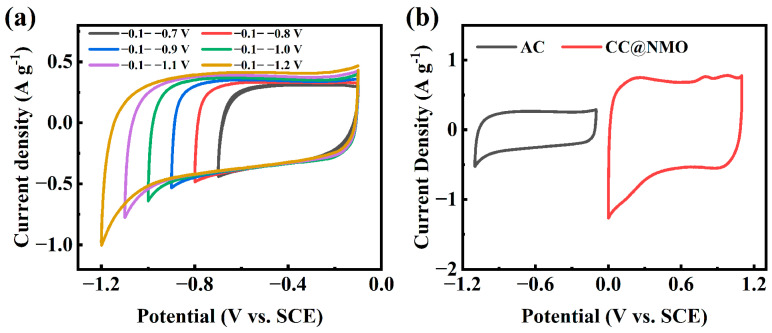
(**a**) CV curves of AC electrode at different potential windows at a 5 mV s^−1^ scan rate; (**b**) CV curves of the cathode and anode at 5 mV s^−1^.

**Figure 6:**
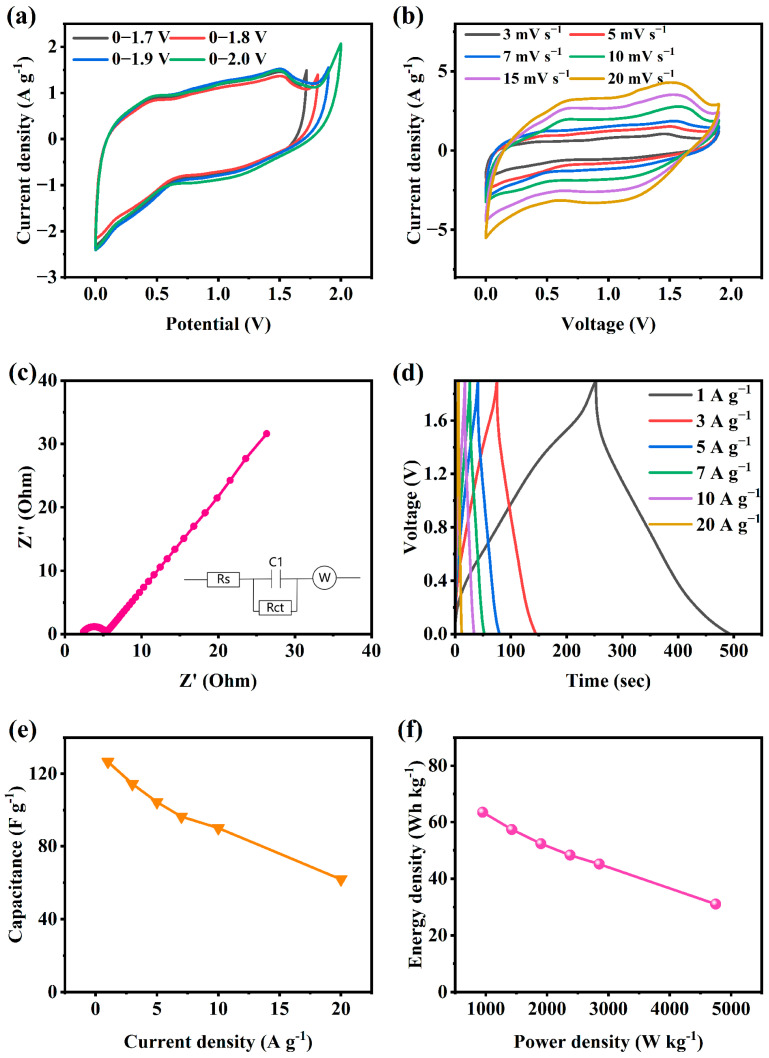
(**a**) CV curves of CC@NMO//AC at different voltage windows, (**b**) CV curves of CC@NMO//AC at different current densities, (**c**) EIS curve of CC@NMO//AC, (**d**) GCD curves of CC@NMO//AC at different current densities, (**e**) specific capacitance of CC@NMO//AC, and (**f**) Ragone plot of CC@NMO//AC.

For further examination of the capacitive behavior and contribution to the energy density of the CC@NMO electrode in ASCs, an aqueous ASC was fabricated. CC@NMO was used as the cathode, while activated carbon (AC) was used as the anode. Before the fabrication, the capacitance of the cathode and anode need to match exactly [17]. Figure 5a shows the CV results of AC in different potential windows at 5 mV s^−1^. The AC electrode works stably from −0.1 to −1.1 V. Figure 5b shows a comparison between CC@NMO and AC at 5 mV s^−1^. Figure S6a shows the GCD curves of the AC electrode at different current densities in −0.1 to −1.1 V. The specific capacitance of AC can be calculated from the GCD curves, as shown in Figure S6b. At 5 A g^−1^, the specific capacitance of AC is 76.75 F g^−1^, which is nearly half of the capacitance of CC@NMO. Obtained by Equation (1), the mass ratio of NMO and AC should be 1:2. The CC@NMO electrode and AC electrode were fabricated into an ASC device (CC@NMO//AC), with 1 M Li_2_SO_4_ as the electrolyte. The electrochemical behavior of CC@NMO//AC is plotted in Figure 6.

Figure 6a shows the CV results of CC@NMO//AC at 5 mV s^−1^. The shape of the curve still retains an ideal rectangular shape at 0–1.9 V, which reflects the electrical double-layer capacitors (EDLCs)’ property of having activated carbon electrodes. Meanwhile, the obvious redox peaks are consistent with the Faraday reaction of the NMO electrodes [44]. Figure 6b displays CV plots of CC@NMO//AC at rising scan rates. Excitingly, the CV shape still maintains its original form when the scan rate rises. The curves do not show significant deformation, and the redox peaks are still obvious at high scan rates, which indicates that the CC@NMO//AC has good capacitive performance and stability [28]. Figure 6c shows the EIS curve of CC@NMO//AC. In the high-frequency region, the shape of the EIS curve is semicircular, while the curve turns to a straight line with a 45° slope in the low-frequency region. The CC@NMO//AC has a small equivalent series resistance Rs, which is only 2.2 Ω. Figure 6d displays the GCD data of the CC@NMO//AC at different current densities. The charging and discharging time can reach up to 490 s at 1 A g^−1^, showing excellent capacitance capacity. Due to the joint action of the EDLC and Faraday capacitor, the shape of the curve is a twisted triangle [17]. As the current density increases, the GCD curve still maintains the approximate triangle, which reveals the good capacitance characteristics and excellent charge/discharge stability of CCNMO//AC. According to Equation (2), the capacitance of CC@NMO//AC can be obtained from the GCD curve. As displayed in Figure 6e, at 1 A g^−1^, the specific capacitance of ASC can reach 126.63 F g^−1^ and can be maintained at 61.8 F g^−1^ at 20 A g^−1^. The relationship between the energy density and power density of CC@NMO//AC can be obtained from a Ragone plot, which can be calculated using Equations (3) and (4). As shown in Figure 6f, the energy density of CC@NMO//AC is up to 63.49 Wh kg^−1^ at 1 A g^−1^, while the power density is 949.8 W kg^−1^. Interestingly, when the current density reaches 20 A g^−1^, the energy density can still remain at 30 Wh kg^−1^.

**Figure 7:**
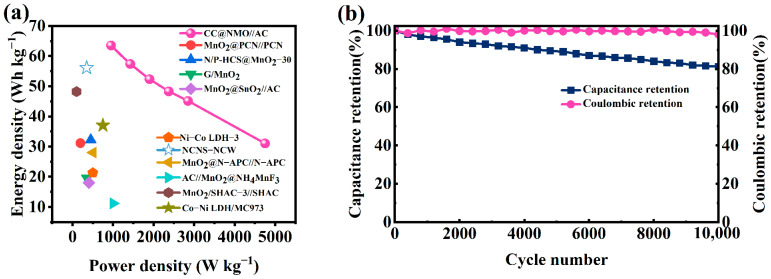
(**a**) Ragone plots of CC@NMO//AC compared to recently reported results; (**b**) cycling stability of CC@NMO//AC at 10 A g^−1^.

Figure 7a shows a comparison between our CC@NMO//AC and other ASC devices mentioned in recently published research articles [45,46,47,48,49,50,51,52,53,54]. Obviously, our CC@NMO//AC outperforms other Mn-based or CC-based devices in terms of energy density. Furthermore, the low cost and simple preparation process needed to synthesize our CC@NMO//AC make our work stand out compared to works about other MnO_2_-based ASCs. Figure 7b displays the long cycling stability of our CC@NMO//AC. After completely cycling for 10,000 times at 10 A g^−1^, the CC@NMO//AC still has 81.2% capacitance retention, which indicates that the device has excellent cycling stability. A comparison of capacitance retention between the CC@MnO_2_//AC and CC@NMO//AC is shown in Figure S7. It can clearly be observed that after 10,000 cycles, the capacitance retention of the CC@MnO_2_//AC decreases quickly, becoming only 74.2%. The structural enhancement of the pre-intercalated NH_4_^+^ ions can significantly reduce the capacity reduction problem caused by structural instability during cycling [33].

**Figure 8:**
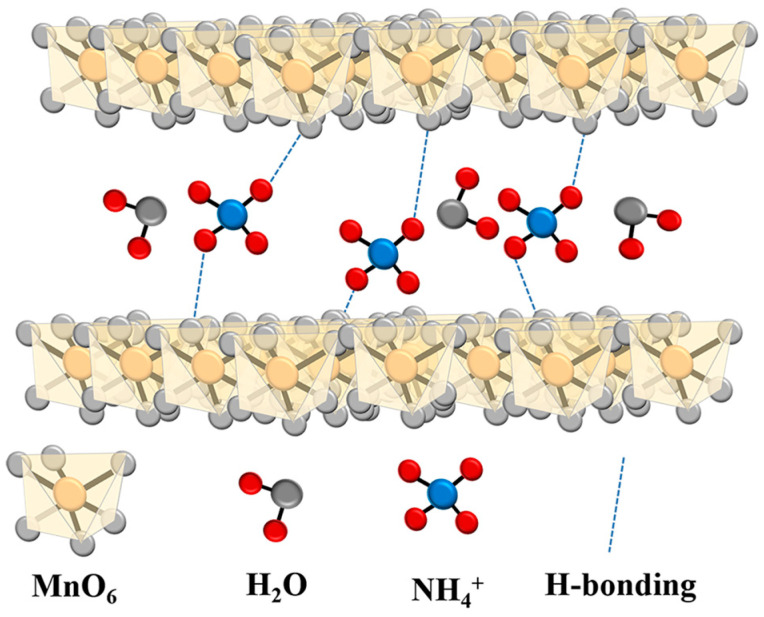
Schematic illustration of CC@NMO.

To further investigate the mechanism of CC@NMO energy storage, there is a need to analyze the microstructure of the material. By analyzing the SEM, XRD, FTIR, and XPS patterns of the CC@NMO, it is possible to obtain the micro-schematic diagram shown in Figure 8, which reveals the storage mechanism of NMO [55]. The ion pre-intercalation influences the electrochemical behavior of MnO_2_ basically through the following three points. Firstly, the pre-intercalated ions can accelerate the diffusion kinetics of MnO_2_-based materials. The guest ions pre-intercalated into the interlayer structure will expand the interlayer spacing to provide more ion diffusion channels [56]. Meanwhile, the constituent water molecules can reduce the effect of the positive ion electrostatic repulsion to accelerate charge and ion transfer [33,57]. Secondly, ion pre-intercalation can stabilize the structure of MnO_2_. Relevant studies have proved that NH_4_^+^ ions provide structural support for the electrode material as structural columns [35,58]. Thirdly, ion pre-intercalation can switch the electrochemical behavior of MnO_2_ into that of a supercapacitor. When ions are pre-intercalated into MnO_2_, the average Mn oxidation state will be reduced, and Mn^3+^/Mn^4+^ redox coupling occurs. More metal ions can be involved in the redox reaction, which makes for a larger pseudo-capacitance. The MnO_2_ will be just like the following equation: M_x_MnO_2_ ⇔ MnO_2_ + xM^+^ + xe^−^ (M^+^ = H^+^, Na^+^, K^+^, NH_4_^+^). The CV curves of ion-pre-intercalated MnO_2_ will increase by a couple of redox peaks [25]. All of the above are consistent with the electrochemical test of CC@NMO//AC. Overall, NH_4_^+^ ion pre-intercalation enhances the electrochemical behavior of the CC@NMO electrode and improves the energy density of ASCs.

## 4. Conclusions

In conclusion, we successfully synthesized a MnO_2_/carbon cloth electrode with NH_4_^+^ ions pre-intercalated by a one-step hydrothermal method. Compared with the CC@MnO_2_ electrode, the CC@NMO electrode obviously has a wider potential window, as well as a larger specific capacity. The pre-intercalation of NH_4_^+^ ion increases the upper limit of the stable working potential window of the electrode from 0.8 V to 1.1 V, and the capacitance can reach 181.4 F g^−1^ at 0.5 A g^−1^. Our improvement of the CC@NMO electrode provides strong support for the high energy density of ASCs. After capacitance matching with an AC electrode, the CC@NMO electrode was fabricated into an ASC. When operating at 1 A g^−1^, the capacitance of the CC@NMO//AC is up to 126.63 F g^−1^. Notably, it still remains at 61.8 F g^−1^ when the current density rises to 20 A g^−1^. Through a Ragone plot, one can see that the energy density of the CC@NMO//AC can reach 63.49 Wh kg^−1^, and the power density can also be up to 949.8 W kg^−1^ at 1A g^−1^. Meanwhile, the pre-intercalation of NH_4_^+^ also significantly enhances the cycling stability. After completely cycling for 10,000 times at 10 A g^−1^, the device still has 81% capacitance retention. The improvement of the stability and energy density of CC@NMO comes from pre-intercalation with NH_4_^+^ ions, which form supportive structures in the interlayer of MnO_2_. Moreover, the NH_4_^+^ ions accelerate charge and ion transfer by enlarging the spacing between layers. Thus, the constructive role of the ammonium ion pre-intercalation technique in improving the electrochemical performance of MnO_2_/carbon cloth electrodes has been demonstrated. This work could prove to be an innovative contribution that helps in increasing the energy density of asymmetric supercapacitors.

## Data Availability

The data presented in this study are available on request from the corresponding author.

## Supplementary Materials

The following supporting information can be downloaded at: https://www.mdpi.com/article/10.3390/ma17081858/s1. Figure S1: (a) TEM image of CC@NMO, (b) HRTEM image of CC@NMO; Figure S2: XRD patterns of CC@NMO, CC@MnO_2_, and CC; Figure S3: EIS curves of CC@NMO and CC@MnO_2_; Figure S4: (a) Comparison of CC@NMO and CC@MnO_2_ at 1 mV s^−1^ at 0–1.1 V and (b) GCD curves of CC@MnO_2_ at 0–0.8 V at different current densities; Figure S5: Cycle stability of CC@NMO at 5 A g^−1^; Figure S6: (a) GCD curves of AC at −0.1 to −1.1 V at different current densities and (b) capacitance of CC@NMO and AC at different current densities; Figure S7: Comparison of cycle stability of CC@NMO//AC and CC@MnO_2_//AC; Table S1: Comparison of energy density and power density with other recent studies.

## References

[1] Larcher D., Tarascon J.M. (2015). Towards greener and more sustainable batteries for electrical energy storage. Nat. Chem..

[2] Yazar S., Arvas M.B., Sahin Y. (2023). S, N and Cl Separately Doped Graphene Oxide/Polyaniline Composites for Hybrid Supercapacitor Electrode. J. Electrochem. Soc..

[3] Khalid M.U., Katubi K.M., Zulfiqar S., Alrowaili Z.A., Aadil M., Al-Buriahi M.S., Shahid M., Warsi M.F. (2023). Boosting the electrochemical activities of MnO_2_ for next-generation supercapacitor application: Adaptation of multiple approaches. Fuel.

[4] Zarshad N., Wu J., Rahman A.U., Ali A., Qiu C., Aisha R., Gao R., Xie Y., Ni H. (2021). Binder and conductive agent-free electrode for the excellent aqueous asymmetrical supercapacitor. Solid State Sci..

[5] Põder K.-S., Eskusson J., Lust E., Jänes A. (2023). Non-Aqueous Zn-ion Hybrid Supercapacitors: Acetonitrile vs Propylene Carbonate Based Electrolyte. J. Electrochem. Soc..

[6] Huang M.C., Sarigamala K.K., Chen H.Y. (2023). Simulation Guided Design of a Hybrid Battery-Type Supercapacitor: A Case Study on MnO@RVC//AC. J. Electrochem. Soc..

[7] Kamila S., Jena B.K., Basu S. (2021). Advances in Electrochemical energy storage device: Supercapacitor. Energy Storage.

[8] Dhibar S., Roy A., Malik S. (2019). Nanocomposites of polypyrrole/graphene nanoplatelets/single walled carbon nanotubes for flexible solid-state symmetric supercapacitor. Eur. Polym. J..

[9] Zarshad N., Rahman A.U., Wu J.H., Ali A., Raziq F., Han L., Wang P., Li G.G., Ni H.M. (2021). Enhanced energy density and wide potential window for K incorporated MnO_2_@carbon cloth supercapacitor. Chem. Eng. J..

[10] Zarshad N., Wu J., Rahman A.U., Tariq M., Ali A., Ni H. (2020). MnO_2_ nanospheres electrode composed of low crystalline ultra-thin nanosheets for high performance and high rate supercapacitors. Mater. Sci. Eng. B-Adv..

[11] Lamba P., Singh P., Singh P., Singh P., Bharti, Kumar A., Gupta M., Kumar Y. (2022). Recent advancements in supercapacitors based on different electrode materials: Classifications, synthesis methods and comparative performance. J. Energy Storage.

[12] Sahoo B.B., Pandey V.S., Dogonchi A.S., Mohapatra P.K., Thatoi D.N., Nayak N., Nayak M.K. (2023). A state-of-art review on 2D material-boosted metal oxide nanoparticle electrodes: Supercapacitor applications. J. Energy Storage.

[13] Lyu L., Seong K.D., Kim J.M., Zhang W., Jin X.Z., Kim D.K., Jeon Y., Kang J., Piao Y.Z. (2019). CNT/High Mass Loading MnO_2_/Graphene-Grafted Carbon Cloth Electrodes for High-Energy Asymmetric Supercapacitors. Nano-Micro Lett..

[14] Ghosh S.K. (2020). Diversity in the Family of Manganese Oxides at the Nanoscale: From Fundamentals to Applications. ACS Omega.

[15] Moniruzzaman M., Kumar Y.A., Pallavolu M.R., Arbi H.M., Alzahmi S., Obaidat I.M. (2022). Two-Dimensional Core-Shell Structure of Cobalt-Doped@MnO_2_ Nanosheets Grown on Nickel Foam as a Binder-Free Battery-Type Electrode for Supercapacitor Application. Nanomaterials.

[16] Aadil M., Mahmood M., Warsi M.F., Alsafari I.A., Zulfiqar S., Shahid M. (2021). Fabrication of MnO_2_ nanowires and their nanohybrid with flat conductive matrix for the treatment of industrial effluents. Flatchem.

[17] Akbar A.R., Saleem A., Rauf A., Iqbal R., Tahir M., Peng G.Q., Khan A.S., Hussain A., Ahmad M., Akhtar M. (2023). Integrated MnO_2_/PEDOT composite on carbon cloth for advanced electrochemical energy storage asymmetric supercapacitors. J. Power Sources.

[18] Zhu Y., Xu H., Chen P., Bao Y., Jiang X., Chen Y. (2022). Electrochemical performance of polyaniline-coated γ-MnO_2_ on carbon cloth as flexible electrode for supercapacitor. Electrochim. Acta.

[19] Wu T.-H., Liang W.-Y., Lin Y.-Q. (2022). Facile synthesis of Cu-intercalated MnO_2_ nanoflakes cathode for enhanced energy storage in zinc-ion batteries. J. Taiwan Inst. Chem. Eng..

[20] Wang S., Ma W., Sang Z., Hou F., Si W., Guo J., Liang J. (2022). Dual-modification of manganese oxide by heterostructure and cation pre-intercalation for high-rate and stable zinc-ion storage. J. Energy Chem..

[21] Chen C., Shi M.J., Zhao Y., Yang C., Zhao L.P., Yan C. (2021). Al-Intercalated MnO cathode with reversible phase transition for aqueous Zn-Ion batteries. Chem. Eng. J..

[22] Zhai X.Z., Qu J., Hao S.M., Jing Y.Q., Chang W., Wang J., Li W., Abdelkrim Y., Yuan H., Yu Z.Z. (2020). Layered Birnessite Cathode with a Displacement/Intercalation Mechanism for High-Performance Aqueous Zinc-Ion Batteries. Nano-Micro Lett..

[23] Wang J., Wang J.-G., Liu H., Wei C., Kang F. (2019). Zinc ion stabilized MnO_2_ nanospheres for high capacity and long lifespan aqueous zinc-ion batteries. J. Mater. Chem. A.

[24] Liu G., Huang H., Bi R., Xiao X., Ma T., Zhang L. (2019). K^+^ pre-intercalated manganese dioxide with enhanced Zn^2+^ diffusion for high rate and durable aqueous zinc-ion batteries. J. Mater. Chem. A.

[25] Chen L., Hao C.Y., Zhang Y.M., Wei Y.R., Dai L.N., Cheng J., Zhang H.Q., Ci L.J. (2021). Guest ions pre-intercalation strategy of manganese-oxides for supercapacitor and battery applications. J. Energy Chem..

[26] Soni S., Pareek K., Jangid D.K., Rohan R. (2020). Carbon cloth-MnO_2_ nanotube composite for flexible supercapacitor. Energy Storage.

[27] Yadav P., Putro D., Kim J., Rai A.K. (2023). Pom-Pom Flower-like Morphology of δ-MnO_2_ with Superior Electrochemical Performances for Rechargeable Aqueous Zinc Ion Batteries. Batteries.

[28] Rahman A.U., Zarshad N., Wu J.H., Shah M., Ullah S., Li G.G., Tariq M., Ali A. (2022). Sodium Pre-Intercalation-Based Na_3_-δ-MnO_2_@CC for High-Performance Aqueous Asymmetric Supercapacitor: Joint Experimental and DFT Study. Nanomaterials.

[29] Patel M.N., Wang X.Q., Wilson B., Ferrer D.A., Dai S., Stevenson K.J., Johnston K.P. (2010). Hybrid MnO_2_-disordered mesoporous carbon nanocomposites: Synthesis and characterization as electrochemical pseudocapacitor electrodes. J. Mater. Chem..

[30] Nam K.W., Kim H., Choi J.H., Choi J.W. (2019). Crystal water for high performance layered manganese oxide cathodes in aqueous rechargeable zinc batteries. Energy Environ. Sci..

[31] Song Y., Pan Q., Lv H.Z., Yang D., Qin Z.M., Zhang M.Y., Sun X.Q., Liu X.X. (2021). Ammonium-Ion Storage Using Electrodeposited Manganese Oxides. Angew. Chem. Int. Ed..

[32] Bu H., Lee H., Hyoung J., Heo J.W., Kim D., Lee Y.J., Hong S.T. (2023). (NH_4_)_2_V_7_O_16_ as a Cathode Material for Rechargeable Calcium-Ion Batteries: Structural Transformation and Co-Intercalation of Ammonium and Calcium Ions. Chem. Mater..

[33] Wang S., Zhao X., Chen H., Guo J., Liu R., Yang D. (2022). Ammonium ion pre-intercalated manganese dioxide with hydrogen bond for high-rate and stable zinc-ion batteries. Ecomat.

[34] Shin M., Sharma K.P., Kim K., Awasthi G.P., Yu C. (2023). The morphology and phase conversion of MnO_2_ in g-CN@MnO_2_ composite with supercapacitor applications. J. Phys. Chem. Solids.

[35] Chen Q., Jin J.L., Kou Z.K., Liao C., Liu Z., Zhou L., Wang J., Mai L.Q. (2020). Zn^2+^ Pre-Intercalation Stabilizes the Tunnel Structure of MnO_2_ Nanowires and Enables Zinc-Ion Hybrid Supercapacitor of Battery-Level Energy Density. Small.

[36] Usui H., Suzuki S., Domi Y., Sakaguchi H. (2020). Impacts of MnO_2_ Crystal Structures and Fe Doping in Those on Photoelectrochemical Charge-Discharge Properties of TiO_2_/MnO_2_ Composite Electrodes. ACS Sustain. Chem. Eng..

[37] Poudel M.B., Lohani P.C., Acharya D., Kandel D.R., Kim A.A., Yoo D.J. (2023). MOF derived hierarchical ZnNiCo-LDH on vapor solid phase grown CuxO nanowire array as high energy density asymmetric supercapacitors. J. Energy Storage.

[38] Wei W.F., Cui X.W., Chen W.X., Ivey D.G. (2011). Manganese oxide-based materials as electrochemical supercapacitor electrodes. Chem. Soc. Rev..

[39] Shao Y.L., El-Kady M.F., Sun J.Y., Li Y.G., Zhang Q.H., Zhu M.F., Wang H.Z., Dunn B., Kaner R.B. (2018). Design and Mechanisms of Asymmetric Supercapacitors. Chem. Rev..

[40] Gou Q.Z., Zhao S., Wang J.C., Li M., Xue J.M. (2020). Recent Advances on Boosting the Cell Voltage of Aqueous Supercapacitors. Nano-Micro Lett..

[41] Jabeen N., Xia Q.Y., Savilov S.V., Aldoshin S.M., Yu Y., Xia H. (2016). Enhanced Pseudocapacitive Performance of α-MnO_2_ by Cation Preinsertion. ACS Appl. Mater. Interfaces.

[42] Zarshad N., Wu J.H., Rahman A.U., Ni H.M. (2020). Fe-MnO_2_ core-shell heterostructure for high-performance aqueous asymmetrical supercapacitor. J. Electroanal. Chem..

[43] Heydari H., Abdouss M., Mazinani S., Bazargan A.M., Fatemi F. (2021). Electrochemical study of ternary polyaniline/MoS_2_-MnO_2_ for supercapacitor applications. J. Energy Storage.

[44] Fu M., Zhu Z.T., Chen W., Yu H., Lv R.T. (2022). Carbon cloth supported flower-like porous nickel-based electrodes boosting ion/charge transfer characteristics of flexible supercapacitors. Carbon.

[45] Feng W.C., Liu G., Wang P.P., Zhou J.W., Gu L.X., Chen L.Z., Li X.Y., Dan Y.Y., Cheng X.F. (2020). Template Synthesis of a Heterostructured MnO_2_@SnO_2_ Hollow Sphere Composite for High Asymmetric Supercapacitor Performance. ACS Appl. Energy Mater..

[46] Li B., Zhang X.H., Dou J.H., Zhang P.X. (2020). Construction of MnO_2_@NH_4_MnF_3_ core-shell nanorods for asymmetric supercapacitor. Electrochim. Acta.

[47] Li D.Y., Lin J., Lu Y., Huang Y., He X., Yu C., Zhang J., Tang C.C. (2020). MnO_2_ nanosheets grown on N-doped agaric-derived three-dimensional porous carbon for asymmetric supercapacitors. J. Alloys Compd..

[48] Tan Y.L., Yang C.X., Qian W.W., Teng C. (2020). Flower-like MnO_2_ on layered carbon derived from sisal hemp for asymmetric supercapacitor with enhanced energy density. J. Alloys Compd..

[49] Wu P.C., Gao M., Yu S.C., Feng M.L., Liu S.H., Fu J.W. (2020). MnO_2_ nanosheets grown on N and P co-doped hollow carbon microspheres for high performance asymmetric supercapacitor. Electrochim. Acta.

[50] Yang Y.K., Niu H., Qin F.F., Guo Z.Y., Wang J.S., Ni G.S., Zuo P.P., Qu S.J., Shen W.Z. (2020). MnO_2_ doped carbon nanosheets prepared from coal tar pitch for advanced asymmetric supercapacitor. Electrochim. Acta.

[51] Zhang M., Zheng H., Zhu H.L., Xu Z.F., Liu R., Chen J.L., Song Q., Song X.J., Wu J., Zhang C.Z. (2020). Graphene-wrapped MnO_2_ achieved by ultrasonic-assisted synthesis applicable for hybrid high-energy supercapacitors. Vacuum.

[52] Wang M., Feng Y., Zhang Y., Li S.S., Wu M.M., Xue L.L., Zhao J.H., Zhang W., Ge M.Z., Lai Y.K. (2022). Ion regulation of hollow nickel cobalt layered double hydroxide nanocages derived from ZIF-67 for High-Performance supercapacitors. Appl. Surf. Sci..

[53] Yang J.J., Li H.L., He S.J., Du H.J., Liu K.M., Zhang C.M., Jiang S.H. (2022). Facile Electrodeposition of NiCo_2_O_4_ Nanosheets on Porous Carbonized Wood for Wood-Derived Asymmetric Supercapacitors. Polymers.

[54] Yao Y.S., Li H.J., Yu Y., Du C., Wan L., Ye H., Chen J., Zhang Y., Xie M.J. (2023). Stabilizing microstructure of Co-Ni layered double hydroxides by magnesium doping and confinement in carbonaceous mesopores for ultrahighly-stable asymmetric supercapacitor. J. Energy Storage.

[55] Mai L.Q., Li H., Zhao Y.L., Xu L., Xu X., Luo Y.Z., Zhang Z.F., Ke W., Niu C.J., Zhang Q.J. (2013). Fast Ionic Diffusion-Enabled Nanoflake Electrode by Spontaneous Electrochemical Pre-Intercalation for High-Performance Supercapacitor. Sci. Rep..

[56] Wang M.Q., Yagi S. (2020). Layered birnessite MnO_2_ with enlarged interlayer spacing for fast Mg-ion storage. J. Alloys Compd..

[57] Sohouli E., Adib K., Maddah B., Najafi M. (2022). Manganese dioxide/cobalt tungstate/ nitrogen-doped carbon nano-onions nanocomposite as new supercapacitor electrode. Ceram. Int..

[58] Yao H.X., Yu H., Zheng Y.Q., Li N.W., Li S., Luan D.Y., Lou X.W., Yu L. (2023). Pre-intercalation of Ammonium Ions in Layered δ-MnO_2_ Nanosheets for High-Performance Aqueous Zinc-Ion Batteries. Angew. Chem. Int. Ed..

