# Supplementary material for: Ammonium Ion-Pre-Intercalated MnO2 on Carbon Cloth for High-Energy Density Asymmetric Supercapacitors
Source: Materials (Basel). 2024 Apr 17;17(8):1858. doi: 10.3390/ma17081858 (PMC11052521; doi:10.3390/ma17081858)
Supplement: Supplementary file 1 [file materials-17-01858-s001.zip › materials-2940169-supplementary.pdf]

Supporting Information

Ammonium ion pre-intercalated MnO_2 on carbon cloth for high energy density asymmetric supercapacitor

Chaoyi Zheng, Xiaohong Sun*, Xinqi Zhao, Xi Zhang, Jiawei Wang, Zhuang Yuan, Zhiyou Gong

Key Laboratory of Advanced Ceramics and Machining Technology of Ministry of Education, School of Materials Science and Engineering, Tianjin University, Tianjin 300072, China

*Corresponding author.

E-mail address: sunxh@tju.edu.cn

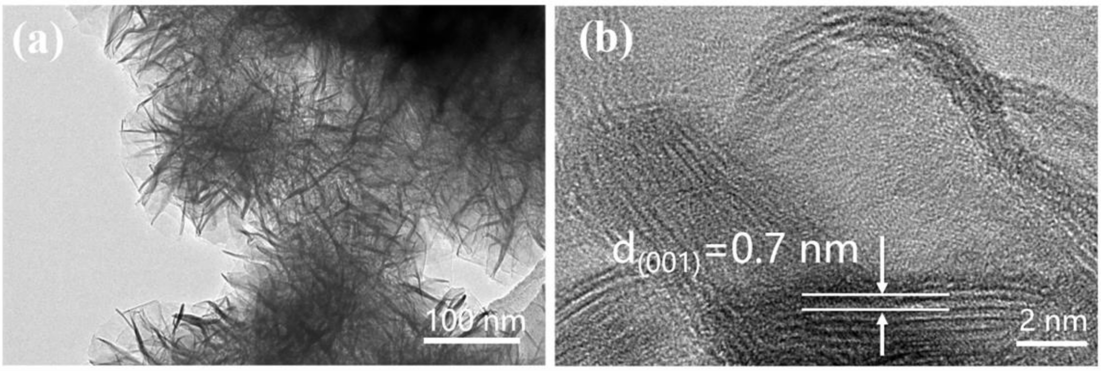

Figure S1. (a) TEM image of CC@NMO, (b) HRTEM image of CC@NMO.

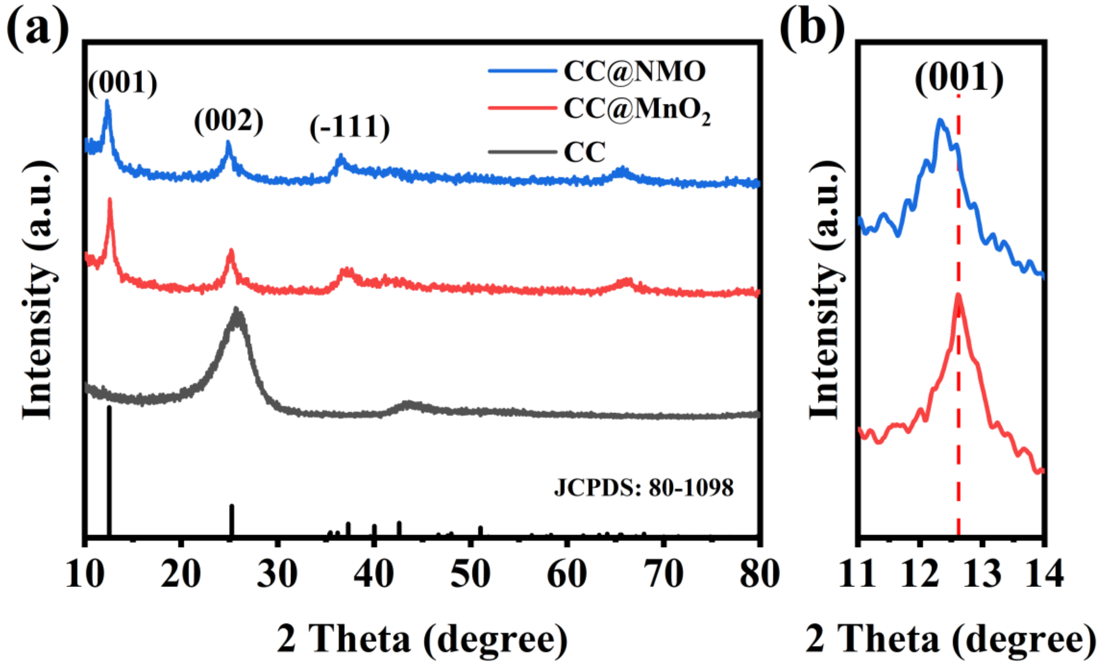

Figure S2. XRD patterns of CC@NMO, CC@MnO_2 and CC.

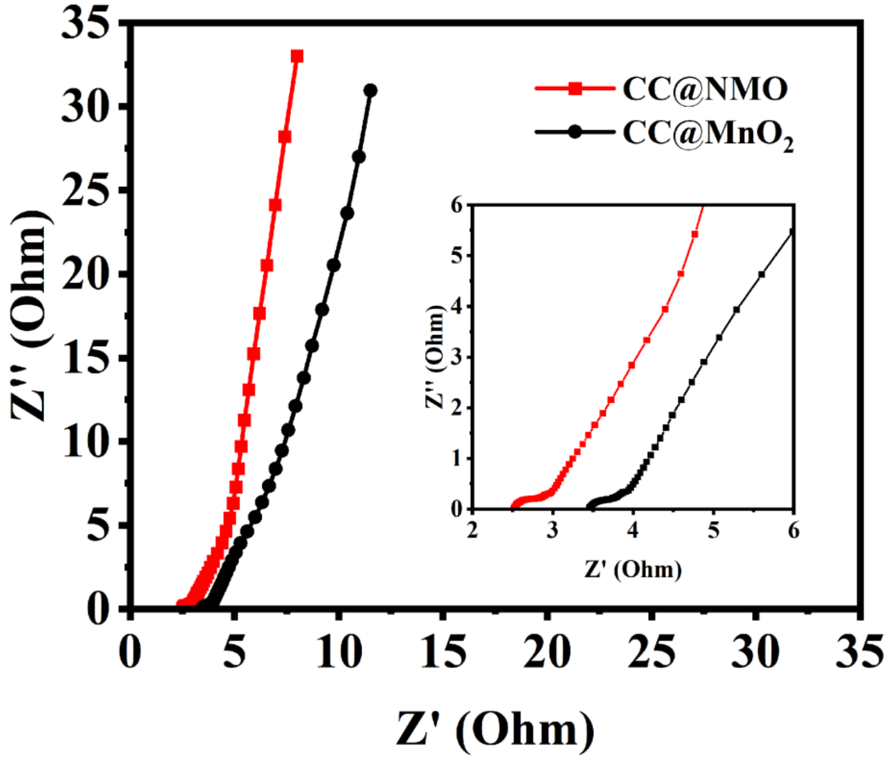

Figure S3. EIS curves of CC@NMO and CC@MnO_2.

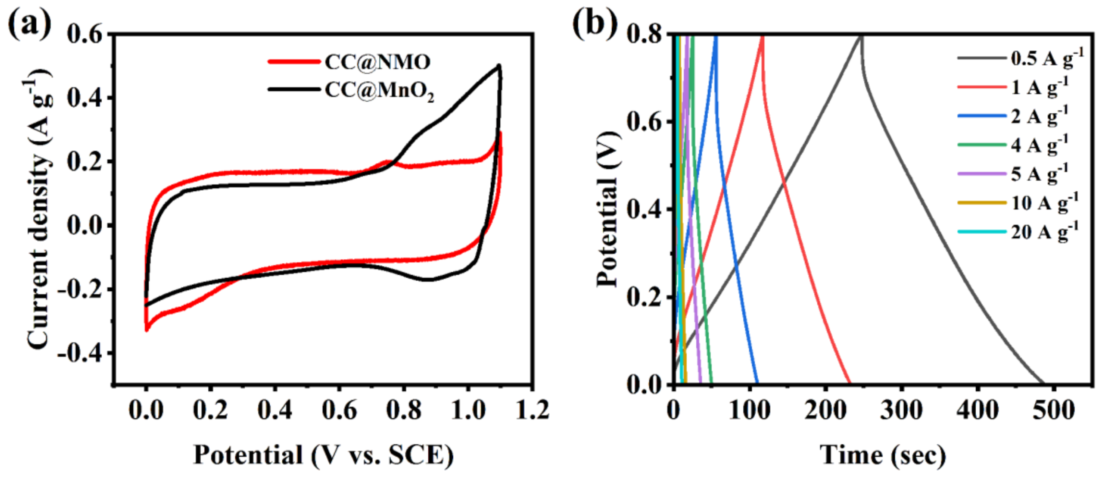

Figure S4. (a) comparison of CC@NMO and CC@MnO_2 at 1 mV s^{-1} at 0-1.1 V, (b) GCD curves of CC@MnO_2 at 0-0.8 V at different current densities.

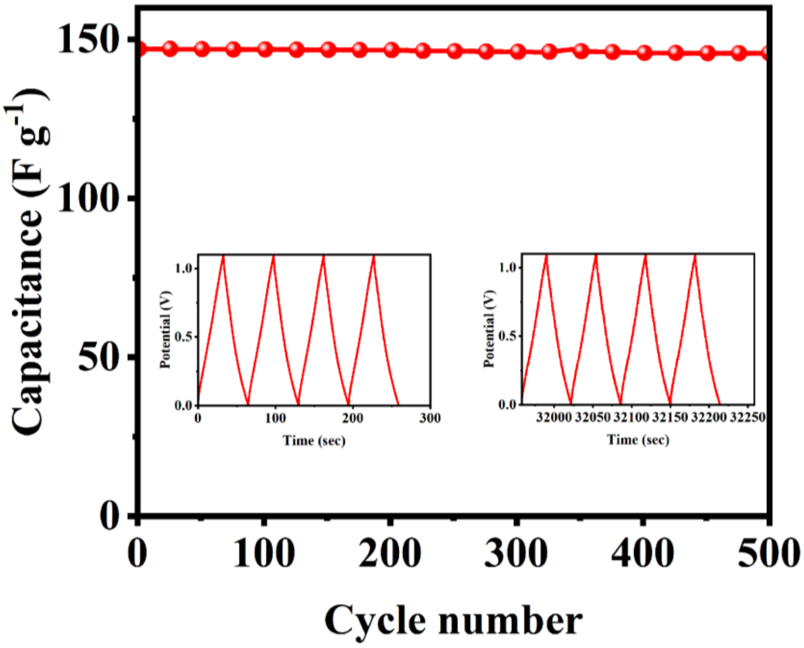

Figure S5. cycle stability of CC@NMO at 5 A g^{-1}.

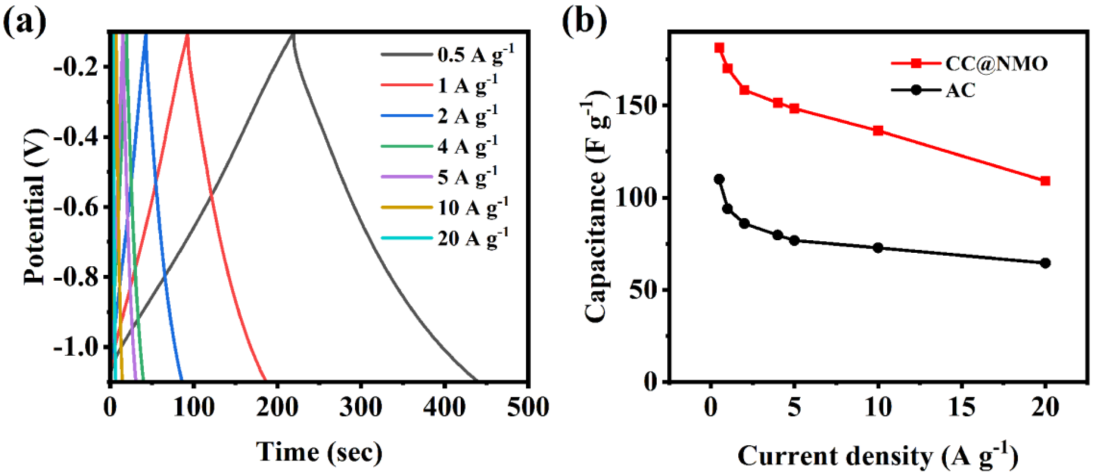

Figure S6. (a) GCD curves of AC at -0.1 to -1.1 V at different current densities, (b) Capacitance of CC@NMO and AC at different current densities.

Table S1. Comparison of energy density and power density with other recent studies.

Sample	Energy density (Wh/kg)	Power density (W/kg)	References
**CC@NMO**	**63.49**	**949.8**	**This work**
MnO_2@PCN//PCN	31.13	193.6	[42]
N/P-HCS@MnO_2-30	32.21	449.8	[43]
G/MnO_2	19.6	351	[44]
MnO_2@SnO_2//AC	18.05	403.6	[45]
MnO_2@N-APC//N-APC	28	506	[46]
AC//MnO_2@NH_4MnF_3	11.2	1000	[47]
MnO_2/SHAC-3//SHAC	48.2	98.5	[48]
Co-Ni LDH/MC973	37	750	[49]
Ni-Co LDH-3	21.28	500	[50]
NCNS–NCW	56.1	349	[51]

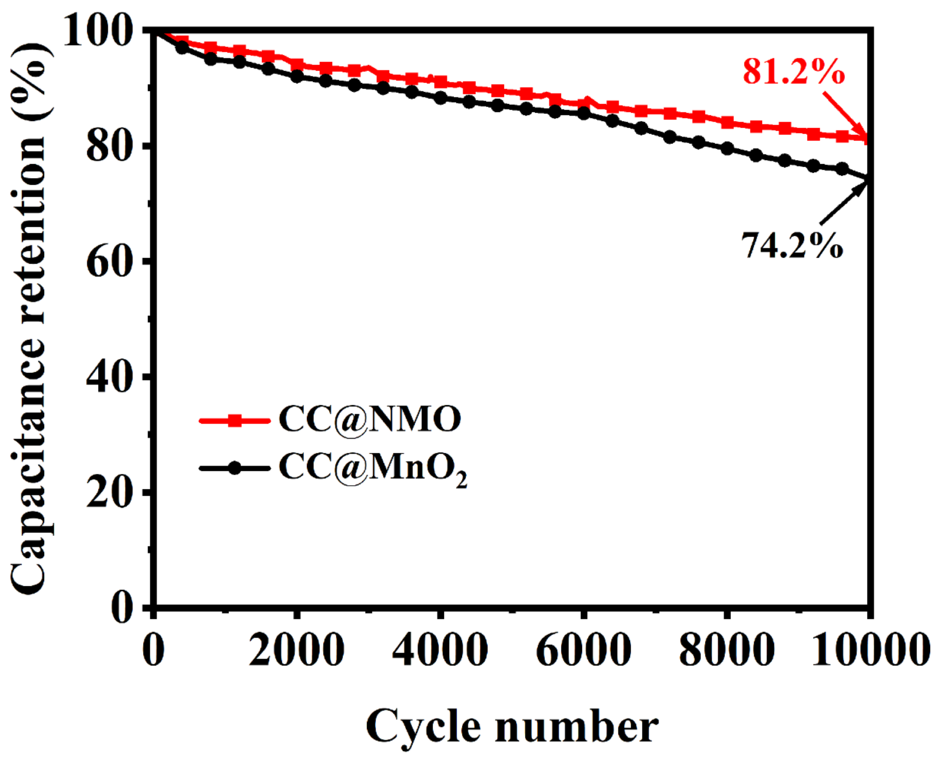

Figure S7. Comparison of cycle stability of CC@NMO//AC and CC@MnO_2//AC.

References

42. Feng, W.C.; Liu, G.; Wang, P.P.; Zhou, J.W.; Gu, L.X.; Chen, L.Z.; Li, X.Y.; Dan, Y.Y.; Cheng, X.F. Template Synthesis of a Heterostructured MnO_2@SnO_2 Hollow Sphere Composite for High Asymmetric Supercapacitor Performance. *ACS Appl. Energy Mater.* **2020**, *3*, 7284-7293, doi:10.1021/acsaem.0c00388.
43. Li, B.; Zhang, X.H.; Dou, J.H.; Zhang, P.X. Construction of MnO_2@NH_4MnF_3 core-shell nanorods for asymmetric supercapacitor. *Electrochim. Acta* **2020**, *347*, doi:10.1016/j.electacta.2020.136257.
44. Li, D.Y.; Lin, J.; Lu, Y.; Huang, Y.; He, X.; Yu, C.; Zhang, J.; Tang, C.C. MnO_2 nanosheets grown on N-doped agaric-derived three-dimensional porous carbon for asymmetric supercapacitors. *J. Alloys Compd.* **2020**, *815*, doi:10.1016/j.jallcom.2019.152344.
45. Tan, Y.L.; Yang, C.X.; Qian, W.W.; Teng, C. Flower-like MnO_2 on layered carbon derived from sisal hemp for asymmetric supercapacitor with enhanced energy density. *J. Alloys Compd.* **2020**, *826*, doi:10.1016/j.jallcom.2020.154133.
46. Wu, P.C.; Gao, M.; Yu, S.C.; Feng, M.L.; Liu, S.H.; Fu, J.W. MnO_2 nanosheets grown on N and P co-doped hollow carbon microspheres for high performance asymmetric supercapacitor. *Electrochim. Acta* **2020**, *354*, doi:10.1016/j.electacta.2020.136681.
47. Yang, Y.K.; Niu, H.; Qin, F.F.; Guo, Z.Y.; Wang, J.S.; Ni, G.S.; Zuo, P.P.; Qu, S.J.; Shen, W.Z. MnO_2 doped carbon nanosheets prepared from coal tar pitch for advanced asymmetric supercapacitor. *Electrochim. Acta* **2020**, *354*, doi:10.1016/j.electacta.2020.136667.
48. Zhang, M.; Zheng, H.; Zhu, H.L.; Xu, Z.F.; Liu, R.; Chen, J.L.; Song, Q.; Song, X.J.; Wu, J.; Zhang, C.Z.; et al. Graphene-wrapped MnO_2 achieved by ultrasonic-assisted synthesis applicable for hybrid high-energy supercapacitors. *Vacuum* **2020**, *176*, doi:10.1016/j.vacuum.2020.109315.
49. Wang, M.; Feng, Y.; Zhang, Y.; Li, S.S.; Wu, M.M.; Xue, L.L.; Zhao, J.H.; Zhang, W.; Ge, M.Z.; Lai, Y.K.; et al. Ion regulation of hollow nickel cobalt layered double hydroxide nanocages derived from ZIF-67 for High-Performance supercapacitors. *Appl. Surf. Sci.* **2022**, *596*, doi:10.1016/j.apsusc.2022.153582.
50. Yang, J.J.; Li, H.L.; He, S.J.; Du, H.J.; Liu, K.M.; Zhang, C.M.; Jiang, S.H. Facile Electrodeposition of $NiCo_2O_4$ Nanosheets on Porous Carbonized Wood for Wood-Derived Asymmetric Supercapacitors. *Polymers* **2022**, *14*, doi:10.3390/polym14132521.
51. Yao, Y.S.; Li, H.J.; Yu, Y.; Du, C.; Wan, L.; Ye, H.; Chen, J.; Zhang, Y.; Xie, M.J. Stabilizing microstructure of Co-Ni layered double hydroxides by magnesium doping and confinement in carbonaceous mesopores for ultrahighly-stable asymmetric supercapacitor. *J. Energy Storage* **2023**, *59*, doi:10.1016/j.est.2022.106422.
